# First-Principles Linear Combination of Atomic Orbitals Calculations of K_2_SiF_6_ Crystal: Structural, Electronic, Elastic, Vibrational and Dielectric Properties

**DOI:** 10.3390/ma17194865

**Published:** 2024-10-02

**Authors:** Leonid L. Rusevich, Mikhail G. Brik, Denis Gryaznov, Alok M. Srivastava, Ilya Chervyakov, Guntars Zvejnieks, Dmitry Bocharov, Eugene A. Kotomin

**Affiliations:** 1 Institute of Solid State Physics, University of Latvia, 8 Kengaraga Str., LV-1063 Riga, Latvia; mikhail.brik@ut.ee (M.G.B.); ilja.cervjakovs@cfi.lu.lv (I.C.); guntars.zvejnieks@cfi.lu.lv (G.Z.); dmitrijs.bocarovs@cfi.lu.lv (D.B.); jevgenijs.kotomins@cfi.lu.lv (E.A.K.); 2 School of Optoelectronic Engineering, CQUPT-BUL Innovation Institute, Chongqing University of Posts and Telecommunications, Chongqing 400065, China; 3 Centre of Excellence for Photoconversion, Vinča Institute of Nuclear Sciences—National Institute of the Republic of Serbia, University of Belgrade, 11000 Belgrade, Serbia; 4 Institute of Physics, University of Tartu, W. Ostwald Str. 1, 50411 Tartu, Estonia; 5 Academy of Romanian Scientists, 3 Ilfov, 050044 Bucharest, Romania; 6 Current Lighting Solutions LLC, 1099 Ivanhoe Road, Cleveland, OH 44110, USA; srivastaam@outlook.com; 7 Transport and Telecommunication Institute, Lauvas Str. 2, LV-1003 Riga, Latvia

**Keywords:** first-principles calculations, DFT, hybrid functionals, atomic and electronic structure, vibrational, dielectric and elastic properties, Raman and IR spectra, K_2_SiF_6_

## Abstract

The results of first-principles calculations of the structural, electronic, elastic, vibrational, dielectric and optical properties, as well as the Raman and infrared (IR) spectra, of potassium hexafluorosilicate (K_2_SiF_6_; KSF) crystal are discussed. KSF doped with manganese atoms (KSF:Mn^4+^) is known for its ability to function as a phosphor in white LED applications due to the efficient red emission from Mn⁴⁺ activator ions. The simulations were performed using the CRYSTAL23 computer code within the linear combination of atomic orbitals (LCAO) approximation of the density functional theory (DFT). For the study of KSF, we have applied and compared several DFT functionals (with emphasis on hybrid functionals) in combination with Gaussian-type basis sets. In order to determine the optimal combination for computation, two types of basis sets and four different functionals (three advanced hybrid—B3LYP, B1WC, and PBE0—and one LDA functional) were used, and the obtained results were compared with available experimental data. For the selected basis set and functional, the above-mentioned properties of KSF were calculated. In particular, the B1WC functional provides us with a band gap of 9.73 eV. The dependencies of structural, electronic and elastic parameters, as well as the Debye temperature, on external pressure (0–20 GPa) were also evaluated and compared with previous calculations. A comprehensive analysis of vibrational properties was performed for the first time, and the influence of isotopic substitution on the vibrational frequencies was analyzed. IR and Raman spectra were simulated, and the calculated Raman spectrum is in excellent agreement with the experimental one.

## 1. Introduction

In modern lighting applications, solid-state white light-emitting diodes (LEDs) are widely used. Among other materials, A_2_XF_6_ (A = Li, Na, K, Rb, Cs; X = Si, Ge, Sn, Ti) fluorite phosphors doped with Mn^4+^ are nowadays considered efficient candidates [1,2,3]. In particular, K_2_SiF_6_:Mn^4+^ is commonly known as a representative material that has been successfully commercialized [4,5,6,7,8,9,10,11,12,13]. This phosphor shows high quantum efficiency, and the narrow red emission bands at 613, 631, 636 and 648 nm are suitable for the human eye [14]. Several theoretical studies were published recently [15,16,17] on the atomic and electronic structure of this phosphor. The first-principles DFT plane wave calculations were focused on the basic K_2_SiF_6_ (KSF) properties using the GGA (PBE) and LDA functionals [15]. This study provided the first estimates of band gap, elastic constants and Debye temperature and examined how structural parameters and band gap change under external hydrostatic pressure. A number of different functionals, namely, LDA [15,16], PBE [15,16,17], GGA [18], SCAN, HSE06, PBE0 and PBE+U [16], were compared using the plane wave basis set with an emphasis on the band gap value. Indeed, a significant problem still remains: the lack of experimental data on the KSF band gap. We can only note the paper [19] where the fundamental absorption edge of pure KSF was determined to occur at ~5.6 eV, which is probably a very underestimated value for the band gap. Based on the experiments for other fluorides [20], the KSF band gap is expected to be about 10 eV.

In this paper, we have performed for the first time detailed first-principles calculations of a wide range of KFS properties using a state-of-the-art quantum chemical approach—a linear combination of atomic orbitals (LCAO) combined with the hybrid functional, as implemented in the CRYSTAL code [21] that is widely used for insulating material modeling [22,23]. Taking into account the practical importance of KSF:Mn^4+^ as a phosphor, it is of significant importance to apply the hybrid functionals here to defectless material first within the formalism of LCAO. It is worth mentioning that the hybrid functionals and Gaussian-type basis sets have been successfully applied to fluorides earlier [24,25], as well as to other wide band gap materials (like diamond, Al_2_O_3_, and MgAl_2_O_4_). Thus, the present study continues our recent DFT consideration of KSF [15] (based on the plane wave basis set and LDA and GGA functionals) by using several DFT functionals, including hybrid and conventional LDA functionals and Gaussian-type basis sets. In this way, we also fill the gap in the application of DFT methods to KSF research. The results for two Gaussian-type basis sets and several DFT functionals for the atomic, electronic and elastic properties are compared with each other and previous plane wave calculations. In addition, calculations of the vibrational frequencies were performed for the first time for this host, to the best of the authors’ knowledge. The comprehensive data obtained in this study are very important for collecting detailed information about prospective phosphor materials, creating a comparative database of crystalline solids used as phosphors and for an assessment of the perspectives for using crystalline solids in lighting applications.

## 2. Materials and Methods

### 2.1. The Equilibrium Structure of KSF

**Figure 1:**
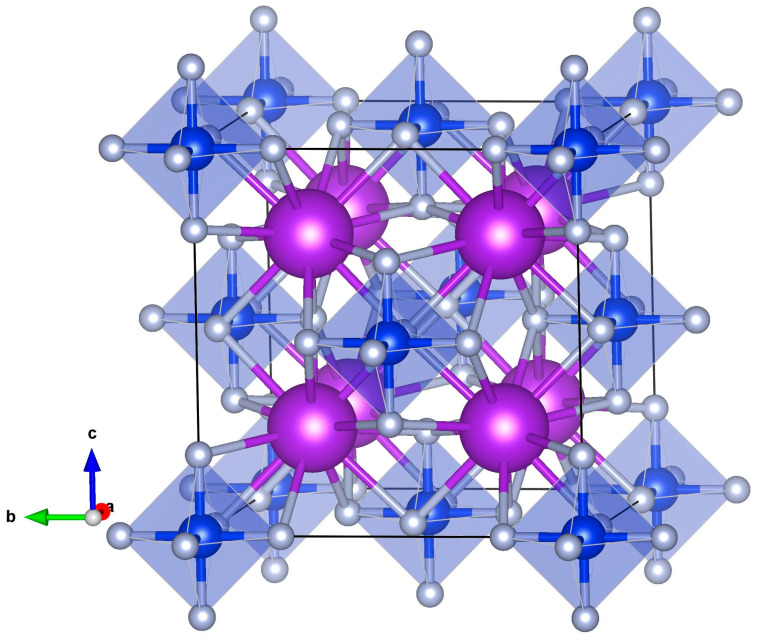
Calculated crystal structure and crystallographic unit cell (36 atoms) of KSF. K atoms—violet balls, Si—blue, F—grey. SiF_6_ octahedra are highlighted. The cube drawn with black lines represents a unit cell.

The perfect KSF has a face-centered cubic crystal structure with *Fm-3m* space group symmetry (space group Nr. 225; SG 225) and experimental lattice constant *a* = 8.134 Å [15]. The crystallographic (conventional) cubic unit cell of this crystal lattice contains 36 atoms, and the Wyckoff positions of atoms are K:8c(1/4, 1/4, 1/4), Si:4a(0, 0, 0) and F:24e(*x*, 0, 0) (after full geometry optimization we obtained *x* = 0.20998). In this structure, K ions are 12-fold coordinated by the fluorine ions, but Si ions are surrounded by 6 fluorine ions, which form a perfect octahedron. Experimental values of K–F and Si–F distances are 2.897 Å and 1.683 Å, respectively [15,26]. A simulated perfect KSF crystal structure with the highlighted crystallographic cubic unit cell is presented in Figure 1. Note that the SiF_6_ octahedra are located at the vertices of the cube and at the centers of its faces.

### 2.2. Computational Details

The first-principles (ab initio) simulations of KSF were performed within the LCAO approximation, as implemented in the CRYSTAL23 computer code [21]. By default, calculations in the CRYSTAL program are performed for a primitive unit cell, which, in the case of KSF, consists of only 9 atoms. This study provides a detailed analysis of the structural, electronic, elastic, vibrational, dielectric, and optical properties of KSF, including simulation of one-phonon Raman and infrared (IR) spectra.

In the beginning, to define an optimal combination of basis set and functional for the calculations, the perfect KSF was simulated using two different basis sets of Gaussian-type functions and four functionals. After that, the results were compared with available experimental data. “Basis set 1” included the following three all-electron basis sets: the basis set for K contains (8s)-(6sp)-(5sp)-(1sp)-(1sp)-(3d) contractions [27] and it is available (like all other basis sets used) on the CRYSTAL Basis Sets Library website [28] as K_86-511G_dovesi_1991 basis set; Si basis set [29] with (8s)-(6sp)-(3sp)-(1sp)-(1sp)-(1d) contractions (Si_86-311G**_pascale_2005); and (7s)-(3sp)-(1sp)-(1sp) F basis set [30] (F_7-311G_nada_1993). The second basis set (“TZVP_2012 basis set”) included the following three all-electron basis sets [31]: (8s)-(4s)-(2s)-(1s)-(1s)-(1s)-(6p)-(3p)-(1p)-(1p)-(1d) K basis set (K_pob_TZVP_2012), (7s)-(3s)-(2s)-(1s)-(1s)-(5p)-(1p)-(1p)-(1p)-(1d) Si basis set (Si_pob_TZVP_2012) and (6s)-(2s)-(1s)-(1s)-(4p)-(1p)-(1p)-(1d) basis set for F (F_pob_TZVP_2012). In preliminary calculations, three global hybrid DFT-HF exchange-correlation functionals (B1WC, PBE0, B3LYP) and one LDA functional were used. The single-parameter B1WC functional [21,32] suggests 16% (by default) of the exact Hartree-Fock (HF) exchange due to improved Wu–Cohen GGA exchange part [33] in combination with the Perdew–Wang PW-GGA correlation functional [21,34]. The PBE0 functional combines the PBE exchange functional with 25% (by default) of HF exchange and the PBE correlation functional [21,35]. The three-parameter B3LYP functional [21,36] combines the BECKE GGA exchange contribution [37], the LDA exchange contribution, the LYP (Lee–Yang–Parr) GGA correlation contribution [38] and the LDA correlation contribution with the exact HF exchange (denoted as *A* parameter in [21]) in the amount of 20% (by default). Additionally, the exchange and correlation parameters are given by *B* = 0.9 and *C* = 0.81 (as is suggested by default in [21], keyword NONLOCAL). The chosen *B* parameter corresponds to the exchange parameter *a_x_* = (1 − *A*)*B* = 0.72 suggested in Becke’s original paper [36]. Finally, the LDA functional was presented, probably, by the most popular LDA formulation—LDA exchange functional and VWN (Vosko–Wilk–Nusair) correlation functional (also known as VWN5) [21]. This LDA formulation is also known as S-VWN.

The five threshold parameters controlling the accuracy of the calculation of the bielectronic Coulomb and HF exchange series (truncation criteria) [21] have been set to 9, 9, 9, 9 and 18. A regular Monkhorst–Pack mesh of points in the reciprocal space with shrinking factor 8 has been used for calculations. The integrations were performed on a default predefined “extra extra large” pruned grid (XXLGRID) consisting of 99 radial points and a maximum of 1454 angular points in the regions relevant for chemical bonding. The Self-Consistent Field (SCF) convergence threshold parameter for the total energy was set to 10^−10^ Hartree (Ha) in all calculations. In order to achieve a very accurate convergence of geometry required for the calculation of vibrational frequencies, during a full geometry optimization, the following convergence criteria were used: RMS (root mean square) of the gradient (TOLDEG) is 0.00003 Ha/Bohr; RMS of the displacement (TOLDEX) is 0.00012 Bohr.

After a comparison of the results obtained with the experimental data, we concluded that the best combination of basis set and functional is the “TZVP_2012 basis set” and B1WC functional (see Section 3). Previous studies have shown that B1WC functional yields reliable results in calculations of different properties of Ti-perovskite crystals and their solid solutions and heterostructures, in particular, the band gaps of these systems [39,40].

The dependence of some selected structural, electronic and elastic properties of KSF on the external hydrostatic pressure (0–20 GPa) was evaluated to see how the pressure-induced changes of the interatomic distances can tune the host’s parameters. The CRYSTAL program implements a procedure that allows the calculation of the elastic properties of crystalline materials, also under pressure [21,41,42]. A complete vibrational analysis was performed for KSF. For the equilibrium geometry, the transverse optical (TO) vibrational frequencies and vibrational contribution to the dielectric tensor were calculated at the Γ-point (in the center of the first Brillouin zone) within the harmonic approximation. In these calculations, the step size of displacement along each Cartesian axis for numerical derivatives (STEPSIZE) was modified to 0.01 Å (0.003 Å by default). The complex dielectric function *ε*(*ν*), depending on the frequency *ν* and associated with the vibrational modes, is the sum of the electronic (high-frequency) and ionic (vibrational) components. It is calculated in CRYSTAL code on the basis of a classical dispersion relation of the Drude–Lorentz model [21,43] and is defined as

(1) $$\varepsilon \left(\nu \right)=\varepsilon_{el}+\sum_{j}\frac{f_{j}\nu_{j}^{2}}{\nu_{j}^{2}-\nu^{2}-i\nu \gamma_{j}}\;\;,$$

where *ε_el_* is the high-frequency dielectric constant, *ν_j_*, *f_j_* and *γ_j_* are the frequency of the *j^th^* TO IR-active vibrational mode, the oscillator strength and the damping factor, respectively [21].

The static dielectric tensor (constant, in our case) *ε*(0) also includes both the electronic and the ionic contributions to the dielectric response. From Equation (1), it is equal to

(2) $$\varepsilon \left(0\right)=\varepsilon_{el}+\sum_{j}f_{j}=\varepsilon_{el}+F\;,$$

where the vibrational contribution *F* is the sum of the oscillator strengths. Wherein the oscillator strengths for an isotropic crystal are computed by means of the expression

(3) $$f_{j}=\frac{4\pi }{V}\frac{Z_{j}^{2}}{\nu_{j}^{2}}\;,$$

where *V* is the cell volume and $Z_{j}^{2}$ is the mass-weighted effective mode Born charge [44].

The electronic contribution *ε_el_* has been calculated in CRYSTAL through the coupled perturbed Hartree–Fock/Kohn–Sham (CPHF/CPKS) scheme adapted for periodic systems [45,46].

The real and imaginary parts of the refractive index *n*(*ν*) are obtained from the complex dielectric function ε(ν) [21]. The integrated intensity *I_n_* of the IR-active vibrational mode *ν_n_* is computed assuming the isotropic response. A few different techniques may be used in the CRYSTAL code for the IR intensities calculation. We have computed IR intensities through both the Berry phase approach, which implies numerical differentiations, and the CPHF approach, which is entirely analytical [21,47]. Both methods give close results, as observed previously in the study of SrTiO_3_, BaTiO_3_ [48] and diamond [49,50] crystals. In turn, the Raman scattering intensities of the Raman-active modes are calculated by a fully analytical method, which is based on the self-consistent solution of first- and second-order CPHF/CPKS equations for the electronic response to external electric fields at the equilibrium geometry [51,52]. Additionally, one-phonon Raman and IR absorbance spectra were simulated using corresponding TO vibrational modes. Both types of spectra were calculated as a convolution of intensities of these modes with the Lorentzian resolution function (FWHM = 8 cm^−1^).

## 3. Results and Discussion

### 3.1. Choice of the Optimal Combination of Basis Sets and Functional

To achieve the most accurate description of KSF vibrational and dielectric properties, it was crucial to carefully select the optimal combination of basis sets and functional based on experimental values of the main structural, electronic and optical parameters. Table 1 and Table 2 present various structural and electronic parameters calculated using two types of basis sets (“Basis set 1” and “TZVP_2012 basis set”) and four different functionals (B1WC, B3LYP, PBE0 and LDA), along with the corresponding available experimental data (“Expt.”). Data in the tables include the following: lattice constant *a*, distances between ions (Si–F, K–F), direct band gap *E_g_*, calculated Mulliken (effective atomic) charges of ions and refractive index *n*, determined as the square root of the real part of the electronic (high-frequency) dielectric constant. Values in parentheses show the difference between calculated and experimental data (relative error in %).

As can be seen from the tables, the structural parameters (lattice constant and distances between ions) are described quite well by all combinations of hybrid functionals and basis sets. Here, the errors do not exceed 2%. The LDA functional describes the structural parameters much worse, which is in agreement with the plane wave calculations [15,16]. Note that absolutely all present simulations slightly overestimate the Si–F distance (a positive sign of the error value in the parentheses). Interestingly, the absolute value and sign of error for *a* and the K–F distance are correlated.

All present calculations give a direct band gap, and all hybrid functionals give a close (within 1 eV) band gap of around 10 eV. In contrast, the LDA functional gives a significantly lower band gap (this is consistent with the results of plane wave calculations [15,16]), as expected. However, our calculated value of the band gap of ~7.9 eV obtained by the LDA functional is even larger than the GGA results of ~7.0 and 7.2 eV in ref. [17] and ref. [18], respectively. Interestingly, the same trend in the band gap for the comparison between the LDA and GGA functionals was already seen in previous plane wave calculations in [15,16]. The present band gap calculated with the B1WC (PBE0) functional (Table 2) is very close to that found with the HSE06 (PBE0) functional and plane wave calculations [16], i.e., 9.73 (10.69) vs. 9.68 (10.44) eV. Due to the lack of reliable experimental data, the calculated band gap value cannot be directly compared with the experiment, but the computations reveal that KSF can definitely be classified as a wide band gap compound. Regarding ion charges, the Si ion charge, calculated using “Basis set 1”, appears to be too small. Moreover, the LDA functional differs from the results of the hybrid functionals for the charges by the fact that the charges of cations are significantly smaller, pointing at the underestimated ionicity of the material. Refractive index *n* is underestimated in all computations with “Basis set 1”, but when using “TZVP_2012 basis set”, the B1WC functional shows the best agreement with the experimental data.

As a result, we conclude that the role of the hybrid functional is crucial for the electronic properties of the material (the band gap value and charges). After analyzing the data in Table 1 and Table 2, we have chosen the B1WC functional and the “TZVP_2012 basis set” as the optimal combination for calculations. All further computations were performed using this combination.

### 3.2. Electronic Density of States (DOS)

**Figure 2:**
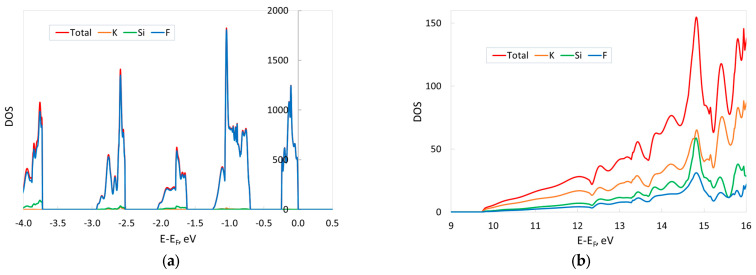
Projected and total electronic DOSs of KSF calculated with the hybrid B1WC functional and “TZVP_2012 basis set” at zero external pressure. Contributions of two K, one Si and six F atoms are shown. The zero-energy value corresponds to the Fermi level. (**a**) Top of valence band (negative values of abscissa axis); (**b**) bottom of conducting band (band gap is 9.73 eV). The number of Legendre polynomials used for the DOS expansion into series is 12 [21].

The calculated electronic density of states (DOS) of KSF, shown in Figure 2, reveals that F ions make an absolutely dominant contribution to the electronic DOS of the valence band (VB), while the conducting band (CB) consists mainly of cation states. Interestingly, at least the top of the VB consists of several more or less narrow (~0.5 eV) sub-bands. The presence of five such sub-bands in the energy range between 0 and −4 eV (Figure 2a) is consistent with the GGA functional and plane wave calculations in Refs. [17,18]. In particular, the two sub-bands lying in deep energy ranges between −2.5 and −4 eV are separated by an almost 0.8 eV gap, whereas the other sub-bands have a separation of ~0.5 eV.

On the other hand, the nature of the Si–F and K–F bonds in KSF could be even better understood from the overlap population analysis. It reveals +0.24|e| and 0.00 |e| (obtained with B1WC) for the overlap population of the Si–F bond and the K–F bond, respectively. These values for the overlap population reflect quite a covalent and ionic nature of these two bonds, respectively. It is interesting to mention that the effective atomic charges of fluorine ions in KSF (Table 2) are smaller in comparison with the more ionic MgF_2_, i.e., −0.59|e| in KSF (B1WC) vs. −0.87|e| in MgF_2_ (the result of PBE0 in Ref. [24]).

### 3.3. Elastic Properties and Effect of External Pressure

**Figure 3:**
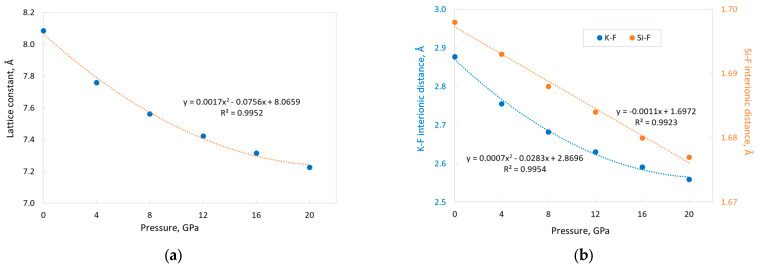
Dependences of the lattice constant (**a**) and the interatomic distances Si–F (right vertical axis) and K–F (left vertical axis) (**b**) on the external pressure with corresponding fitting (dotted lines). Calculations were performed with “TZVP_2012 basis set” and the B1WC functional.

Table 3 and Table 4 present the dependences of selected structural and electronic parameters, as well as some elastic constants of KSF, on external hydrostatic pressure. Data from Table 3 reveal that the structural parameters of KSF (lattice constant *a* and distances between ions) decrease monotonically with increasing pressure. Both K–F and *a* dependence can be approximated by second-order polynomials, wherein the linear fit provides sufficient precision for description (with more or less the same accuracy) of the pressure effect on the Si–F interionic distance (Figure 3). Notice that the change in the interionic distance is one order of magnitude smaller for Si–F than for K–F. As expected, the application of external pressure leads to a decrease in the lattice constant and interionic distances. However, it is important to pay attention to the rate of decrease of these parameters. When the pressure changes from 0 to 20 GPa, the lattice constant *a* and Si–F and K–F distances decrease by 10.6%, 1.2% and 11.1%, respectively. This result shows the high stiffness of the Si–F bond and that the decrease in unit cell volume with pressure occurs mainly due to a reduction in the size of the KF_12_ polyhedra. Note that as the pressure increases, the relative position of the F ion in the unit cell changes. This is indicated by the variation in the non-dimensional free *x* coordinate of the F ion in the Wyckoff position 24e of the crystal lattice. This coordinate increases monotonically with pressure (Table 3), which means that a relative (in fractional units) Si–F interionic distance will increase, while K–F will decrease.

**Figure 4:**
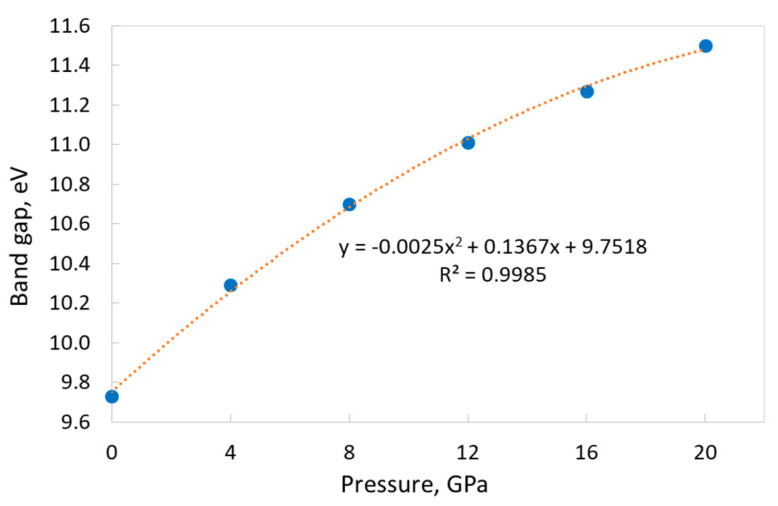
Dependence of the KSF band gap value on the external pressure with corresponding fitting (orange dotted line). “TZVP_2012 basis set” and the B1WC functional.

The band gap monotonically increases with increasing pressure (see Table 3), which is quite expected for a direct band gap. It is a non-linear dependence, and it can be described by a second-order polynomial (Figure 4). The increase in band gap value is 18.2% at the pressure change from 0 to 20 GPa.

Note that the use of a fitting (Figure 3 and Figure 4) allows for interpolating the corresponding parameters at any pressure value in the studied range.

The Mulliken charges (their absolute values) of the K and F ions decrease monotonically with increasing pressure, whereas the charge of the Si ion remains relatively constant (Table 3). It is also in line with the smaller change in the interionic distance for the Si–F bond compared to the K–F bond with the pressure, as discussed above. The bond character remains unchanged with the pressure for the Si–F bond (the overlap population is +0.25 |e| at 20 GPa) as well as for the K–F bond (only a small negative value of −0.02 |e| appears at 20 GPa for the K–F bond from the overlap population analysis).

Table 4 demonstrates the pressure influence on the KSF elastic properties. The symmetric elastic tensor of KSF (as a cubic crystal system) has only three independent constants: *C_11_*, *C_12_* and *C_44_*. These constants, as well as the bulk modulus (*B*), Hill shear modulus (*G*), Young modulus (*E*) and Poisson ratio (*ν*), are presented in Table 4. It is important to note that the Hill shear modulus is defined as the average of the Voigt (an upper limit for *G*) and Reuss (a lower limit for *G*) shear moduli [15,17], and the Voigt and Reuss bulk moduli of KSF are equal to each other. Our values of the elastic constants *C_11_* and *C_12_*, as well as the bulk modulus *B* (Table 4, the hybrid B1WC functional), calculated at zero pressure, are slightly larger than those calculated in plane wave calculations with the GGA functional in Refs. [15,17]. The same situation is seen with the constants *G*, *E* and *ν* if we compare our results (Table 4) and the results in ref. [15] (*E* and *ν*) and ref. [17] (shear modulus *G*). At the same time, the elastic constant *C_44_* in our calculations (Table 4) is somewhat smaller compared to the results of GGA calculations in refs. [15,17]. Under an external pressure of 20 GPa, all elastic constants *C_ij_*, as well as the moduli *B* and *G*, are larger in calculations with the hybrid B1WC functional (Table 4) than with the GGA functional [17]. An inspection of Table 4 clearly shows that all elastic constants increase with increasing pressure. The authors of Ref. [17], who calculated, in particular, three elastic constants and bulk and shear moduli depending on pressure, made similar conclusions.

### 3.4. Vibrational Properties and IR and Raman Spectra

As a primitive unit cell of KSF consists of nine atoms, there are 27 normal lattice vibrations at the Γ-point of the Brillouin zone (BZ) of this system. Three of them are acoustic vibrations, and the other twenty-four are optical vibrations, which are distributed over nine phonon modes. The TO modes with the corresponding frequencies calculated in this study are presented in Table 5. It is clear from the table that the set of phonon modes is given by F_1g_ + 2F_2g_ + 3F_1u_ + F_2u_ + E_g_ + A_1g_, i.e., it includes seven triply degenerate modes (F-modes), one doubly degenerate mode (E_g_) and one non-degenerate mode (A_1g_). Three F_1u_ modes are IR-active, four modes (2F_2g_ + E_g_ + A_1g_) are Raman-active and two modes (F_1g_ and F_2u_) are silent (neither Raman- nor IR-active). Interestingly, the IR- and Raman-active modes are strictly separated (no one mode is both IR- and Raman-active). All calculated TO vibrational modes are located in the range of 70–755 cm^−1^ (2.1–22.6 THz).

**Figure 5:**
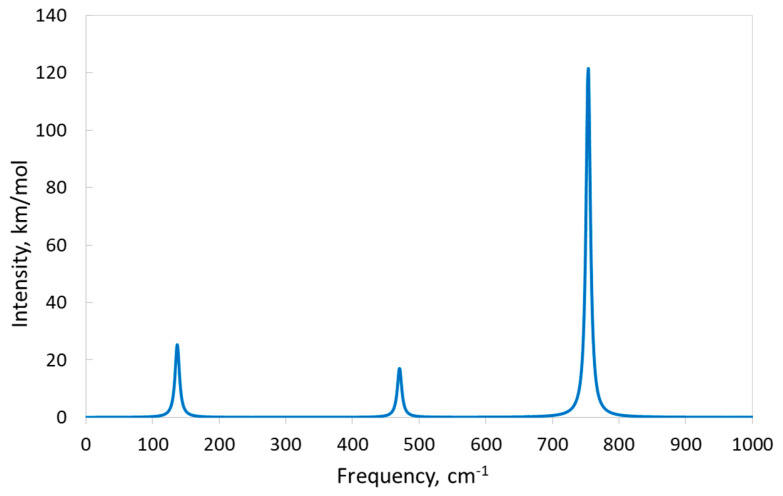
One−phonon IR absorbance spectrum of KSF simulated with the B1WC functional and “TZVP_2012 basis set”.

**Figure 6:**
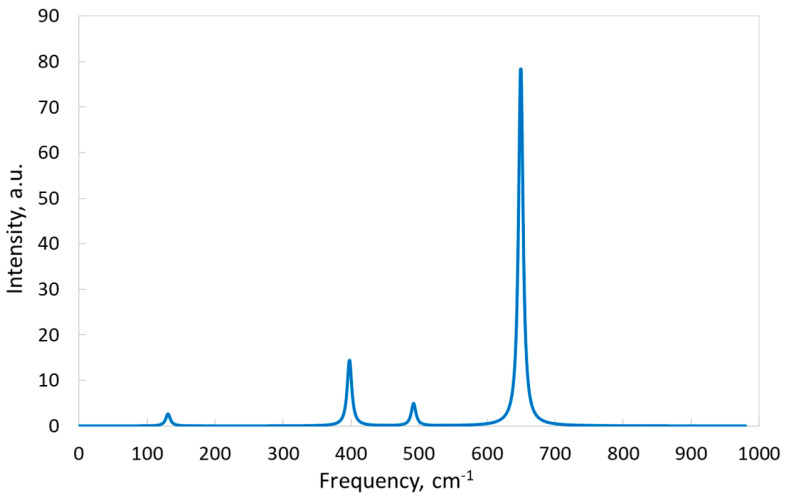
Raman spectrum of KSF simulated with the B1WC functional and “TZVP_2012 basis set”.

The relevant simulated one-phonon IR and Raman spectra are presented in Figure 5 and Figure 6, respectively. The three IR-active modes are well separated by frequency (Table 5), so each peak in the IR spectrum is generated by only one vibrational mode, and there are three distinct peaks in the spectrum (Figure 5). The most intensive peak corresponds to the 754 cm^−1^ F_1u_ TO phonon mode. The analysis of atom displacements in this mode reveals that Si atoms demonstrate the maximal displacements; moreover, these atoms take part in vibrations along all axes. At the same time, each fluorine atom participates in vibrations, mainly along only one axis, but the contribution of K atoms to this mode is negligible. On the contrary, K atoms give the main contribution to the first peak (137 cm^−1^) of the IR spectrum, although the contributions of Si and F atoms are also significant. All atoms vibrate in this mode along all axes. Finally, the middle peak (471 cm^−1^) arises due to vibrations of F and Si atoms along all axes. K atoms practically do not vibrate in this mode.

To check the conclusions derived from the analysis of the atom displacements in the vibrational modes, we have used the option of isotopic substitution (the possibility of modifying the atomic masses of specific atoms), implemented in the CRYSTAL code [21]. We have compared the vibrational frequencies computed using standard atomic masses and those obtained with new relative isotopic masses. Such analysis gives the possibility of evaluating the main contributions of the selected atoms to the vibrational modes. In general, an increase in atomic masses leads to a decrease in vibrational frequencies, but this decrease depends on the contribution of a corresponding atom (group of atoms) to the particular vibrational mode. We have used this technique previously when investigating the point defects in diamonds [49,50]. In the current study, we have increased the relative masses of atoms in KSF by approximately 10%, and the isotopic shifts of vibrational mode frequencies were calculated for three different cases: (i) relative atomic mass of the Si atom is changed from 28 to 31; (ii) masses of all K atoms are changed from 39 to 43; and (iii) masses of all F atoms are changed from 19 to 21.

As a result, returning to the IR-active modes, we conclude that isotopic substitution fully confirms the estimates obtained from the atom displacement analysis. The isotopic shift of mode 754 cm^−1^ is −23.2 cm^−1^ in the case of the isotopic substitution of the Si atom, −13.2 cm^−1^ for the change of F atom masses and only a few thousandths cm^−1^ (practically negligible) in the case of K atoms. The isotopic shift of mode 471 cm^−1^ is −4.6 cm^−1^ for Si atom isotopic substitution, −19.1 cm^−1^ for F atoms and again, a few thousandths cm^−1^ for K atoms. The lowest frequency IR mode (137 cm^−1^) has an isotopic shift of −0.4 cm^−1^ for Si atom substitution, −2.0 cm^−1^ for F atoms and −4.2 cm^−1^ for K atoms. Thus, vibrations of Si and F atoms contribute to all IR modes, but K atoms vibrate only in one mode (137 cm^−1^), and their contribution to the other two modes can be neglected.

As in the case of the IR spectrum, each peak of the Raman spectrum (Figure 6) is also formed by only one vibrational mode since the Raman modes are well separated by frequency (Table 5). Interestingly, both Raman-active and silent modes are associated with vibrations of one type of atom (either K or F). Let us first consider the Raman-active modes. Relative intensities of Raman lines (they are normalized to the most intensive line) corresponding to Raman-active modes are 0.034:0.183:0.062:1 (for the lines with frequencies 131, 398, 492 and 649 cm^−1^, respectively). These four modes generate four peaks in the Raman spectrum (Figure 6). One peak is dominant (649 cm^−1^).

It is noteworthy that Si atoms remain stationary in all Raman-active modes. Vibrations of K atoms along all axes produce the 131 cm^−1^ mode. The isotopic shift of this mode for K atom isotopic substitution is −6.3 cm^−1^, whereas the isotopic shift for F atoms is less than one thousandth cm^−1^. On the contrary, the 398 cm^−1^ mode arises from vibrations of F atoms, and the isotopic shift due to K atoms is less than one thousandth cm^−1^. The vibrations of each F atom in this mode occur in one plane, and the isotopic shift due to F atoms is −19.4 cm^−1^. Exclusively, vibrations of F atoms form the 492 and 649 cm^−1^ modes (Si and K atoms do not vibrate at all in these modes). At the same time, each F atom vibrates along only one axis.

Below, we compare our simulated Raman spectrum with the experimental spectra measured for KSF:Mn^4+^, namely KSi_0.95_Mn_0.05_F_6_ (measured at ambient pressure and room temperature) in [53] and for the Mn^4+^-dilute KSF system and undoped KSF (measured at room temperature) in [54]. The experimental spectrum for SiF_6_ vibrations in KSF:Mn^4+^ consists of three characteristic lines at ~405 (410), ~475 (480) and ~655 (658) cm^−1^, with a dominant peak at ~655 (658) cm^−1^ in ref. [53] and ref. [54] given in parentheses. These frequency values are very close to the frequencies of the calculated modes that form the simulated Raman spectrum (see Table 5 and Figure 6). The comparison between the spectra for pure KSF and KSF:Mn^4+^ in [54] did not reveal significant differences for the three characteristic lines, as discussed here. There is, however, one more line at ~120 cm^−1^ in [53] and 131 cm^−1^ in Table 5 and Figure 6. Overall, our calculated results for the Raman spectra completely coincide with the results of the experiments. In its turn, this suggests that the vibrations of F atoms entirely (for lines 492 and 649 cm^−1^, see Table 5) or almost entirely (for line 398 cm^−1^, see Table 5) form the corresponding peaks in the simulated Raman spectrum (see paragraph above).

The silent modes are also formed by vibrations of F atoms. In these modes, each F atom vibrates in one plane. Si and K atoms remain stationary in silent modes. The isotopic shifts for F atom isotopic substitution are −3.5 cm^−1^ (mode 72 cm^−1^) and −12.9 cm^−1^ (mode 264 cm^−1^).

### 3.5. Dielectric and Optical Properties

The static dielectric tensor *ε*(0) given by Equation (2) is a constant in the case of defectless KSF since the three diagonal elements of the second rank tensor are equal and the off-diagonal elements are zero. The estimation of *ε*(0) was performed in two steps. Firstly, the high-frequency contribution *ε_el_* was calculated, containing only the electronic response. After that, the vibrational contribution *ε_vib_* to the static dielectric tensor was computed during the calculation of TO vibrational frequencies. Herewith, *ε_vib_* is the sum of the vibrational contributions of each IR-active mode. Our calculations give, for the electronic component, also called the optical dielectric constant, the value *ε_el_* = 1.745. Typically, *ε_el_* is not the main source of a possible high *ε*(0). The large static dielectric constant usually arises from the lattice (vibrational) contribution. Our computations reveal that the sum of the vibrational contributions of three IR-active modes to the static dielectric constant is *ε_vib_* = 2.628, which is quite comparable to the electronic contribution *ε_el_*. The main contribution to the *ε_vib_* (2.160 from 2.628) comes from the vibrational mode at 137 cm^−1^. This mode is the lowest frequency vibrational mode among the modes contributing to the static dielectric constant. This result is consistent with Equation (3), where oscillator strength is inversely proportional to the square of the frequency. Thus, for perfect KSF, our calculations yield a static dielectric constant of *ε*(0) = 4.373.

**Figure 7:**
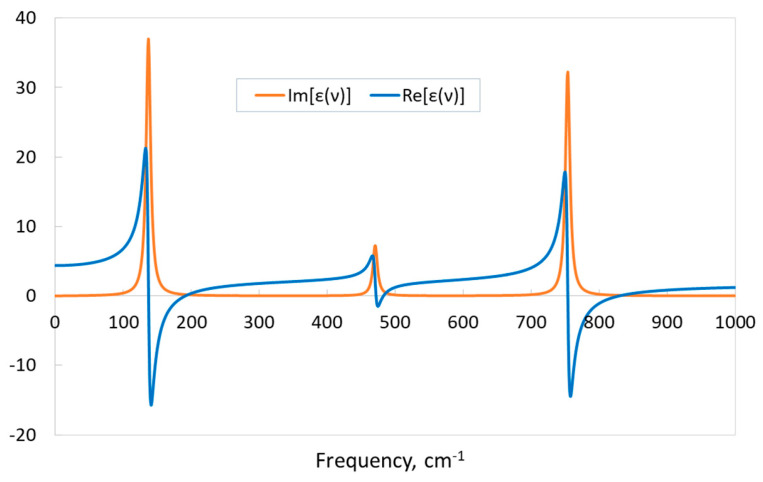
Real Re[*ε*(*ν*)] and imaginary Im[*ε*(*ν*)] parts of KSF relative permittivity. “TZVP_2012 basis set” and the B1WC functional.

In general, permittivity is a complex function with an imaginary part that is associated with conductivity, which determines absorption. The CRYSTAL code allows the calculation of the real and imaginary parts of the complex dielectric function *ε*(*ν*) depending on the vibrational frequency *ν*. The maxima of the imaginary part of the dielectric function correspond to the frequencies of the TO vibrational modes. Figure 7 exhibits the real and imaginary parts of the calculated dielectric function of KSF. Three absorption bands are observed, corresponding to three IR-active vibrational modes of the material. In these regions, Im[*ε*(*ν*)] has maxima, and Re[*ε*(*ν*)] drops sharply. Note that the real part of the dielectric function at *ν* = 0, Re[*ε*(*0*)] is the static dielectric constant *ε*(0), and the presence of negative values of Re[*ε*(*ν*)] indicates that the electromagnetic waves of the corresponding frequencies will not propagate in the medium but will be reflected from its boundary.

**Figure 8:**
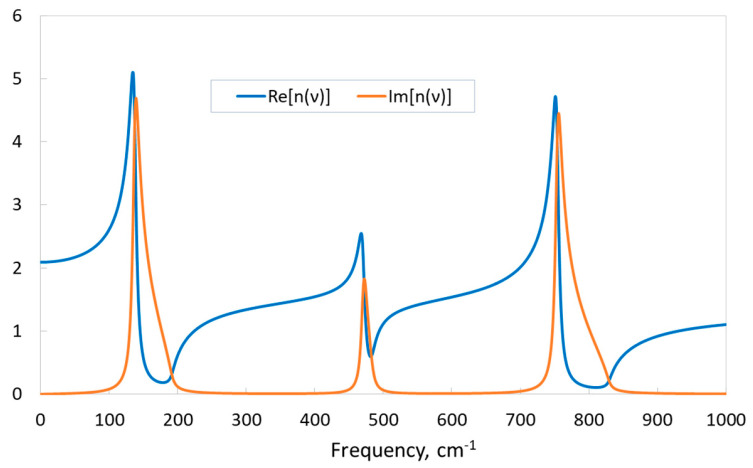
Real Re[*n*(*ν*)] and imaginary Im[*n*(*ν*)] parts of KSF refractive index. “TZVP_2012 basis set” and the B1WC functional.

The refractive index, calculated from the high-frequency (electronic) contribution *ε_el_* to the static dielectric constant, is 1.321, which is in very good agreement with the experimental data (see Table 2). A complex frequency-dependent refractive index *n*(*ν*) is used to describe the propagation of electromagnetic waves in absorbing materials. In this context, the imaginary part is responsible for attenuation, and the real part is responsible for refraction. The real and imaginary parts of such a complex refractive index of KSF are presented in Figure 8. Similar to the dielectric function, the imaginary part Im[*n*(*ν*)] has three maxima corresponding to the IR-active modes of KSF. Far from these areas, in frequency ranges where the imaginary part is close to zero and the medium is transparent for electromagnetic waves, the real part of the refractive index Re[*n*(*ν*)] increases slightly with frequency (normal dispersion). However, it decreases rapidly in the region of absorption bands (anomalous dispersion). Note that Re[*n*(*0*)] is equal to the square root of the static dielectric constant *ε*(0), and possible values of the real part of the refractive index below unity indicate that the phase speed of light of certain frequencies in this medium can exceed the speed of light in a vacuum.

### 3.6. Debye Temperature Evaluation

In the last subsection, we estimate the elastic wave velocities in a crystal and Debye temperature from the obtained elastic constants. The longitudinal *v_l_* and transverse *v_t_* velocities of elastic waves can be calculated [15,17,55] as

(4) $$v_{l}=\sqrt{\frac{3B+4G}{3\rho }}\;,\;\;\;\;\;\;\;v_{t}=\sqrt{\frac{G}{\rho }\;,}$$

where *B* is the bulk modulus, *G* is the shear modulus and *ρ* is the material density. Using *v_l_* and *v_t_* from (4), it is possible to calculate the average elastic wave velocity (an effective sonic velocity) *v_m_* weighted by the number of polarization states [15,17,56]:

(5) $$v_{m}={\left[\frac{1}{3}\left(\frac{2}{v_{t}^{3}}+\frac{1}{v_{l}^{3}}\right)\right]}^{-1/3}.$$

Finally, using *v_m_* from (5), Debye temperature *Θ_D_* is calculated by the formula [15,17,57]

(6) $$\Theta_{D}=\frac{h}{k}{\left(\frac{3nN_{A}}{4\pi }\frac{\rho }{\mu }\right)}^{1/3}v_{m}\;,$$

where *h* and *k* are Planck’s and Boltzmann’s constants, respectively, *N_A_* is Avogadro’s number, *μ* is the molecular weight, and *n* denotes the number of atoms per formula unit (nine for KSF).

Thus, using the values of *B* and *G* moduli from Table 4 and Equations (4)–(6), elastic wave velocities and Debye temperature can be calculated as functions of pressure. It is important to take into account in computations the dependence of density on pressure since density increases by 40% when pressure changes from 0 to 20 GPa. The results of these calculations are summarized in Table 6. The obtained data demonstrate that velocities and Debye temperature, like the density of crystal, increase with increasing pressure. Comparing our results with previous calculations [17], we can conclude that both the velocities and (especially) the Debye temperatures coincide quite well at 0 GPa. However, the rate of growth of all these parameters in our calculations is slightly higher. Unfortunately, due to the lack of experimental data, the results of calculations cannot be compared with the experiments, even at atmospheric pressure.

## 4. Conclusions

The first-principles calculations of a wide range of the structural and physical properties of the perfect KSF crystal were performed in the present paper. When doped with Mn^4+^ ions, KSF is widely used as a commercial red phosphor in white LEDs. Several computational settings were used to determine the one whose results best agreed with the experimental data. We applied the hybrid DFT functionals and Gaussian-type basis sets for the study of KSF. Specifically, we found differences in the lattice parameters, interionic distances and electronic band gaps between conventional methods like LDA, GGA and hybrid B1WC functional. The effects of the hydrostatic pressure were successfully modeled by optimizing the crystal structure and performing calculations at elevated pressures between 0 and 20 GPa. The dependencies of the lattice constant, interionic distances and electronic and elastic properties on pressure were obtained and fitted to linear or second-order polynomial functions, which allows for reliable estimations of all those parameters at any value of pressure in the studied range. In particular, the Si–F bond turned out to be stiffer than the K–F bond. Also, the bond properties are discussed within the Mulliken population analysis. Calculations of the Raman, IR and silent vibrational modes were performed. Not only the frequencies of the normal modes but also the character of motion of all relevant atoms and the influence of the isotopic substitution on the vibrational frequencies were analyzed and described. Excellent agreement was found between the simulated and experimentally measured Raman spectra from the literature. Evaluations of the Debye temperature and elastic wave velocities are discussed. The comprehensive dataset obtained in this study provides a valuable reference for the properties of the KSF crystal and can be used in modeling impurities such as Mn^4+^ within this material.

## Figures and Tables

**Table 1:** Lattice constant *a*, interionic distances (Si–F, K–F), band gap *E_g_*, Mulliken (effective atomic) charges of ions and refractive index *n* of KSF, calculated using “Basis set 1” and four functionals (B1WC, B3LYP, PBE0, LDA) at zero external pressure in comparison with corresponding experimental data (“Expt.”).

	B1WC	B3LYP	PBE0	LDA	Expt. [15]
*a*, Å	8.134 (0.00%)	8.263 (1.59%)	8.197 (0.77%)	7.818 (−3.88%)	8.134
Si–F, Å	1.704 (1.25%)	1.715 (1.90%)	1.706 (1.37%)	1.701 (1.07%)	1.683
K–F, Å	2.895 (−0.07%)	2.942 (1.55%)	2.918 (0.72%)	2.776 (−4.18%)	2.897
*E_g_*, eV	9.83	10.03	10.84	7.88	
F, |e|	−0.410	−0.455	−0.430	−0.370	
Si, |e|	0.534	0.807	0.649	0.320	
K, |e|	0.964	0.961	0.966	0.950	
*n*	1.264 (−5.67%)	1.249 (−6.79%)	1.249 (−6.79%)	1.326 (−1.04%)	1.34

**Table 2:** Lattice constant *a*, interionic distances (Si–F, K–F), band gap *E_g_*, Mulliken (effective atomic) charges of ions and refractive index *n* of KSF, calculated using “TZVP_2012 basis set” and four functionals (B1WC, B3LYP, PBE0, LDA) at zero external pressure in comparison with corresponding experimental data (“Expt.”).

	B1WC	B3LYP	PBE0	LDA	Expt. [15]
*a*, Å	8.086 (−0.59%)	8.200 (0.81%)	8.153 (0.23%)	7.796 (−4.16%)	8.134
Si–F, Å	1.698 (0.89%)	1.709 (1.54%)	1.700 (1.01%)	1.696 (0.77%)	1.683
K–F, Å	2.877 (−0.69%)	2.919 (0.76%)	2.903 (0.21%)	2.768 (−4.45%)	2.897
*E_g_*, eV	9.73	9.96	10.69	7.90	
F, |e|	−0.585	−0.605	−0.604	−0.539	
Si, |e|	1.807	1.919	1.899	1.679	
K, |e|	0.852	0.856	0.861	0.778	
*n*	1.321 (−1.42%)	1.305 (−2.61%)	1.305 (−2.61%)	1.381 (3.06%)	1.34

**Table 3:** Dependence of KSF lattice constant *a*, non-dimensional free *x* coordinate of the F ion (Wyckoff position 24e in SG *Fm-3m*), interionic distances (Si–F, K–F), band gap *E_g_* and Mulliken (effective atomic) charges of ions on external pressure. “TZVP_2012 basis set” and the B1WC functional were used for calculations.

	0 GPa	4 GPa	8 GPa	12 GPa	16 GPa	20 GPa
*a*, Å	8.086	7.759	7.563	7.424	7.316	7.227
*x*	0.20998	0.21814	0.22322	0.22685	0.22969	0.23201
Si–F, Å	1.698	1.693	1.688	1.684	1.680	1.677
K–F, Å	2.877	2.755	2.682	2.630	2.591	2.559
*E_g_*, eV	9.73	10.29	10.70	11.01	11.27	11.50
F, |e|	−0.585	−0.575	−0.569	−0.566	−0.563	−0.561
Si, |e|	1.807	1.802	1.803	1.807	1.811	1.817
K, |e|	0.852	0.824	0.807	0.793	0.783	0.774

**Table 4:** Effect of pressure on the elastic constants (*C_11_*, *C_12_*, *C_44_*), bulk modulus *B*, Hill shear modulus *G*, Young modulus *E* and Poisson ratio *ν* of KSF. “TZVP_2012 basis set” and the B1WC functional.

	0 GPa	4 GPa	8 GPa	12 GPa	16 GPa	20 GPa
*C_11_*, GPa	36.95	61.82	84.65	107.39	129.47	151.39
*C_12_*, GPa	14.94	34.14	52.10	69.63	86.52	102.94
*C_44_*, GPa	12.74	20.11	26.95	34.20	41.51	49.12
*B*, GPa	22.28	43.36	62.95	82.22	100.83	119.09
*G*, GPa	12.02	17.31	22.01	26.95	31.86	36.98
*E*, GPa	30.56	45.83	59.14	72.88	86.48	100.54
*ν*	0.27	0.32	0.34	0.35	0.36	0.36

**Table 5:** Calculated transverse optical (TO) vibrational modes at the Γ-point of the KSF Brillouin zone (BZ). Letters A and I in the third column indicate whether the mode is, respectively, active or inactive for IR and Raman scatterings. “TZVP_2012 basis set” and the B1WC functional.

Modes	Frequencies, cm^−1^	IR–Raman
F_1g_	72	I–I
F_2g_	131	I–A
F_1u_	137	A–I
F_2u_	264	I–I
F_2g_	398	I–A
F_1u_	471	A–I
E_g_	492	I–A
A_1g_ *	649	I–A
F_1u_	754	A–I

* Note that this mode is denoted as A_g_ in the CRYSTAL output file.

**Table 6:** Dependences of KSF density *ρ*, the elastic wave velocities (*v_l_*, *v_t_*, *v_m_*) in crystal and Debye temperature *Θ_D_* on pressure. The data in this table were obtained using results of first-principles calculations with “TZVP_2012 basis set” and the B1WC functional.

	0 GPa	4 GPa	8 GPa	12 GPa	16 GPa	20 GPa
*ρ*, kg/m^3^	2763	3126	3377	3570	3730	3869
*v_l_*, m/s	3723	4610	5228	5753	6198	6597
*v_t_*, m/s	2086	2353	2553	2748	2923	3092
*v_m_*, m/s	2321	2637	2868	3090	3289	3480
*Θ_D_*, K	282	334	373	409	442	473

## Data Availability

The original contributions presented in the study are included in the article, further inquiries can be directed to the corresponding authors.
